# Generalization of Stochastic Visuomotor Rotations

**DOI:** 10.1371/journal.pone.0043016

**Published:** 2012-08-14

**Authors:** Hugo L. Fernandes, Ian H. Stevenson, Konrad P. Kording

**Affiliations:** 1 Department of Physical Medicine and Rehabilitation, Northwestern University and Rehabilitation Institute of Chicago, Chicago, Illinois, United States of America; 2 PDBC, Instituto Gulbenkian de Ciência, Oeiras, Portugal; 3 Instituto de Tecnologia Química e Biológica, Universidade Nova de Lisboa, Oeiras, Portugal; 4 Redwood Center for Theoretical Neuroscience, University of California, Berkeley, California, United States of America; 5 Department of Physiology, Northwestern University, Chicago, Illinois, United States of America; 6 Department of Applied Mathematics, Northwestern University, Chicago, Illinois, United States of America; University of Sussex, United Kingdom

## Abstract

Generalization studies examine the influence of perturbations imposed on one movement onto other movements. The strength of generalization is traditionally interpreted as a reflection of the similarity of the underlying neural representations. Uncertainty fundamentally affects both sensory integration and learning and is at the heart of many theories of neural representation. However, little is known about how uncertainty, resulting from variability in the environment, affects generalization curves. Here we extend standard movement generalization experiments to ask how uncertainty affects the generalization of visuomotor rotations. We find that although uncertainty affects how fast subjects learn, the perturbation generalizes independently of uncertainty.

## Introduction

A central goal of systems neuroscience in general and motor control research in particular is to understand how sensorimotor behaviors, such as reaching, are represented and learned. One factor that regularly influences movement planning and execution is uncertainty. For example, when we grasp objects our hands move very differently depending on our level of uncertainty; if we are uncertain about an object’s position, we open our hands wider, move more slowly and approach the object with our hands aligned with the direction of highest uncertainty [1]. This example highlights the fact that variability in the external world affects behavior and suggests that uncertainty must be represented in the nervous system.

Many studies in the field of motor control have used generalization experiments to examine the neural representation of movement, asking how learning a perturbation in one task affects behavior on novel tasks [2], [3], [4], [5], [6], [7], [8], [9], [10], [11], [12], [13], [14]. By studying which aspects of the behavior are transferred between tasks and which tasks a behavior transfers to, these experiments have investigated how we represent and modify movement and task variables. Generalization is sensitive to many factors including the coordinate system, nature, and complexity of the perturbation [5], [7], [11], [12], movement variables such as speed [8] and posture [9], as well as the extent and type of training and feedback [10], [15]. However, one factor that has not yet been studied in the context of generalization experiments is uncertainty. Many studies have explored how uncertainty affects behavior [1], [16], [17], [18], but how uncertainty influences generalization has received little attention.

From a normative viewpoint, subjects should generalize what they have learned about a perturbation in one situation to a novel situation only if they expect the perturbation to occur in the novel situation. Behavior in novel situations reveals what subjects expected to occur, and these expectations may be affected by several factors including task similarity or familiarity with the type of perturbation. It has been difficult to formalize this normative approach to generalization, since natural movement statistics and natural perturbation statistics are difficult to collect. However, any normative description of generalization must take uncertainty into account, since variability in the external world can have strong effects on behavior; task uncertainty [17], sensory uncertainty [19] and motor noise [20], [21], have all been shown to affect individual movements and learning, and may affect the similarity between movements as well as the resulting generalization.

A common interpretation of generalization from one task to another is that stronger generalization indicates a larger overlap in the neural representations of the two tasks. For instance, Krakauer et al. [12] measured generalization of planar, center-out reaching movements with rotation and gain perturbations. Training with a rotational perturbation in one direction produced strong generalization to nearby angular targets, but did not affect movements to novel targets with large angular separations from the training direction (>45°). On the other hand, visuomotor gain perturbations tended to generalize globally, to all reach directions. This finding suggests that the internal neural representation that changed in response to these perturbations is activated during movements to similar angular directions, and that there may be a polar representation of planar reaches, where reach angle and extent are independent. Here we extend a visuomotor rotations experiment of Krakauer et al. [12] by introducing variability in the perturbations.

It is not clear, a priori, if and how uncertainty might influence generalization. One hypothesis, from a normative perspective, might be that task variability will make subjects more conservative and generalization narrower. High variability may indicate to subjects that it is less likely that the perturbation will be present for novel targets. A second hypothesis is that higher uncertainty will result in broader neural representations and that these could be reflected in wider generalization patterns. Several theories of the neural representation of uncertainty explicitly predict that uncertainty changes neural tuning. In particular, these models predict that tuning of individual neurons becomes wider with higher uncertainty [22], [23], and there is some experimental data suggesting that this may be the case [24], [25] (see Discussion). If generalization patterns trivially reflect overlapping neural tuning and if neural tuning becomes wider with increasing uncertainty then we might expect generalization to become broader with increasing uncertainty. However, it is difficult to match behavioral results to precise neural mechanisms; generalization between two movements can typically only be interpreted in terms of the degree of behavioral similarity between the movements or in terms of an abstract similarity between the neural representations of the two movements [4], [26], [27], [28].

Here, with the goal of examining how uncertainty influences generalization patterns, we designed an experiment to manipulate the mean and the variance of noisy visuomotor rotations relative to the central starting position while subjects performed center-out reaches. We examined how subjects adapt to distributions of perturbations applied during movement in one direction (training direction). On each trial a rotation sampled from a Gaussian distribution with fixed mean and variance was applied to a hidden cursor controlled by the subjects’ index finger. After the subjects adapt, we measure how the learned mean generalizes to movements into other directions. The mean perturbation remained the same under the different noise conditions. If this mean is the only factor driving generalization movements, then we would not expect to see any difference between generalization curves. On the other hand, since uncertainty has been shown to affect many different types of movement, it is important to test whether or not generalization changes under noisy perturbations. We found that the mean of the perturbation generalizes with a width of about 30 degrees, in line with previous studies [12], [29], [30]. We found that the variance of the perturbation changes the speed and extent of learning, but, importantly, generalization is unaffected.

## Results

**Figure 1:**
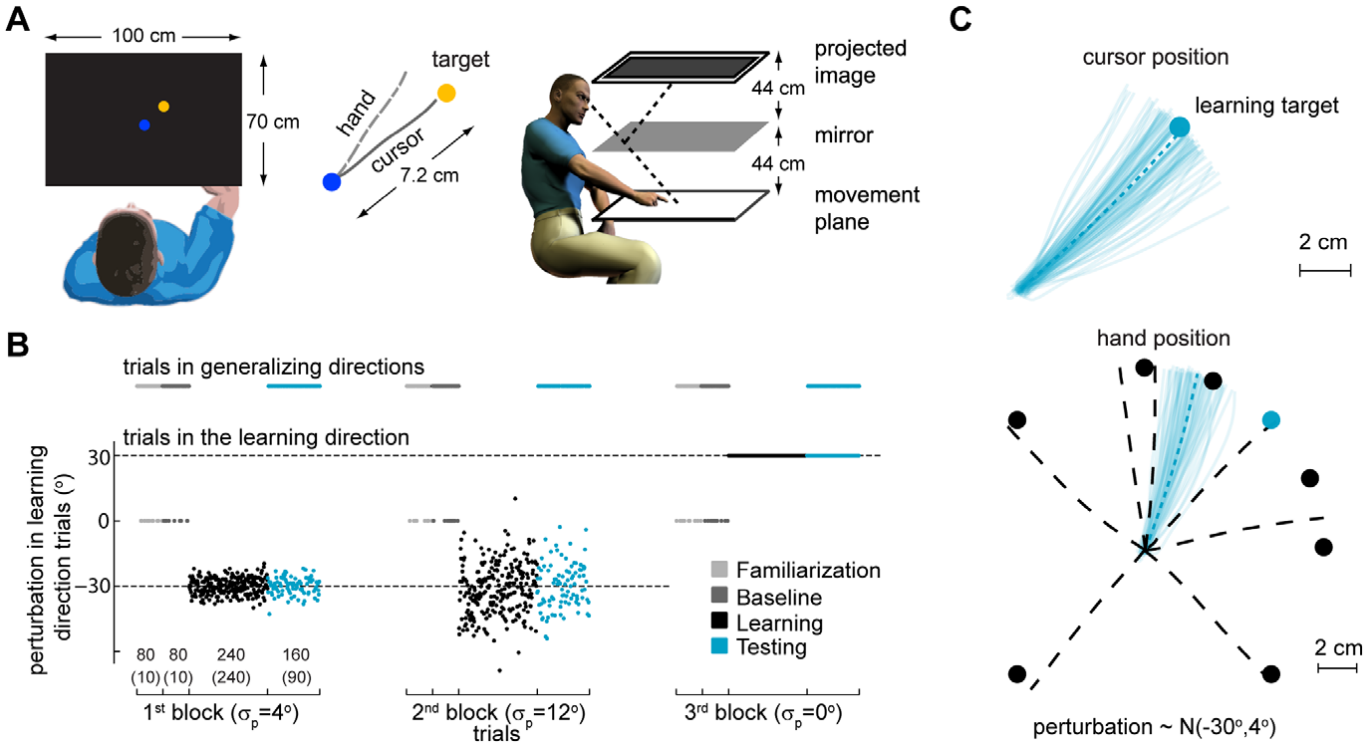
Experimental setup, protocol and typical trajectory data. **A**) Experimental setup. Subjects control the position of a hidden cursor on the screen with their right index finger. A projector-mirror system allows the image on-screen to be perceived as being in the movement plane. Subjects were incentivized to reach to a target (yellow) starting from a central target position (blue). The experiment assesses generalization of the learned mean under different uncertainty conditions. **B**) Perturbations and block design for an individual subject. Sequence of trials in the learning direction and generalizing directions and perturbations applied to trials in the learning direction for an individual subject. 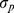 denotes the standard deviation of the distribution of perturbations. Each block is composed of 4 sub-blocks: *familiarization, baseline, learning* and *testing*. Numbers in the 1^st^ block horizontal axis correspond to the total number of trials during each sub-block (no brackets) and the number of trials towards the learning direction during each sub-block (between brackets). **C**) Typical hand and cursor position during a *testing* sub-block. Thin colored lines are movements towards the learning target (colored circles). Dashed thick lines are average hand position for reaches in each direction. Black circles are targets in generalizing directions.

Here we ask how a perturbation that varies randomly across trials is learned for one direction and how adaptation to this perturbation affects movements into other directions. We thus extend movement generalization studies by analyzing how uncertainty, induced by variability or noise in the perturbation, affects generalization patterns. Subjects controlled the position of a hidden cursor with their right index finger by making planar reaches in a projector-mirror system that blocked the view of the hand (**Fig. 1A**). They made center-out reaches from the workspace center to one of eight targets while a visuomotor rotation, relative to the workspace center position, was applied to the hidden cursor position. The visuomotor rotation was drawn randomly each trial from a Gaussian distribution with fixed mean and variance (**Fig. 1B** **)**. During *learning* subjects were incentivized to make reaches to one of the targets and received endpoint feedback about the cursor position that allowed them to adapt to the perturbations. During *testing* subjects made reaches to the other targets, without endpoint feedback, allowing us to examine the generalization patterns (**Fig. 1C**). We then measured how learning about the rotations under different variance conditions generalized.

**Figure 2:**
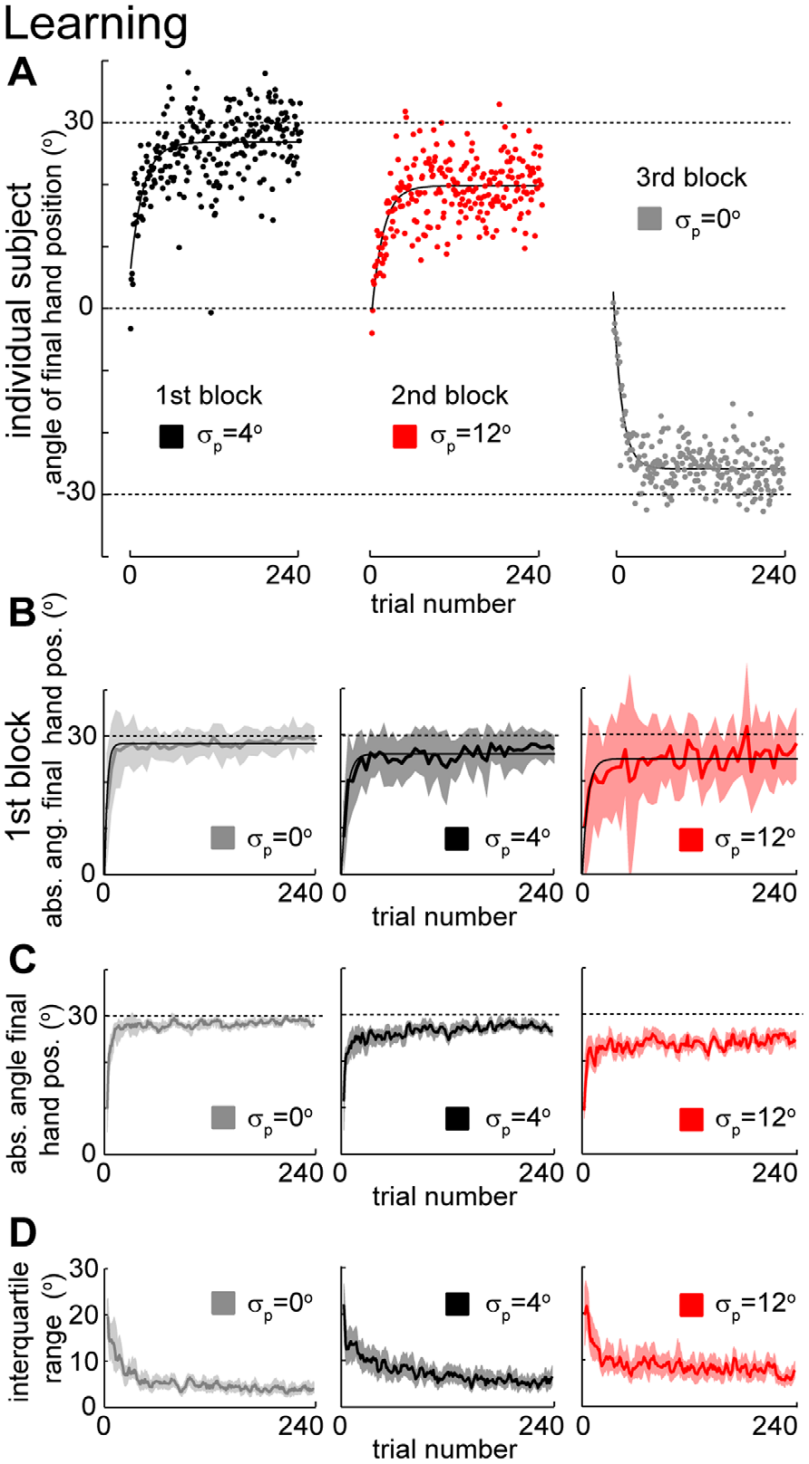
Learning of mean under different variance conditions. **A**) Learning the mean of a perturbation during the first perturbation block for a typical subject. Solid lines denote exponential fits. **B**) Learning the mean of a perturbation during the first perturbation block across subjects (n = 8, n = 4, and n = 4 for the standard deviations, 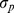 of 0°, 4° and 12°, respectively). Thick lines are average (±SD) across subjects considering bins of 5 trials. Thin lines are exponential fits. Grey dashed lines indicate the absolute average of the imposed perturbation (30°). **C**) Learning the mean of a perturbation considering all blocks for each variance condition. Thick lines denote medians across subjects and trials in a trial window of 5 trials. Shaded area is 95% confidence region (bootstrap). **D**) Variability of angle of final hand position. Thick lines denote the interquartile range of the angle of final hand position across subjects and a trial window of 5 trials. Shaded area is 95% confidence region (bootstrap).

Subjects (n = 16) were confronted with a rotational perturbation that caused the cursor to deviate from the true hand position as subjects moved away from the center of the workspace. We presented three blocks of training with the same absolute mean perturbation (±30 degrees) but different variability (standard deviations, 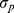: 0°, 4° or 12°). Since the sign of the mean of the perturbation was randomly chosen for each subject and condition, in order to compare across subjects we transformed the measure of generalization so that positive hand position angles always refer to hand position angles that counteract the average perturbation – we call this measure the *absolute angle of final hand position*. In agreement with previous studies [31], [32], we found that subjects rapidly adapt to the mean rotation, and, while they initially make large errors, subjects learn to counter-act the perturbation so that errors become small over the course of a few trials (**Fig. 2**). We found that learning is fastest (p<0.03, bootstrap) and most complete (p<0.001, bootstrap) for the condition with zero variance (see Methods for details). As the uncertainty of the perturbation increased learning was both slower and less complete.

**Figure 3:**
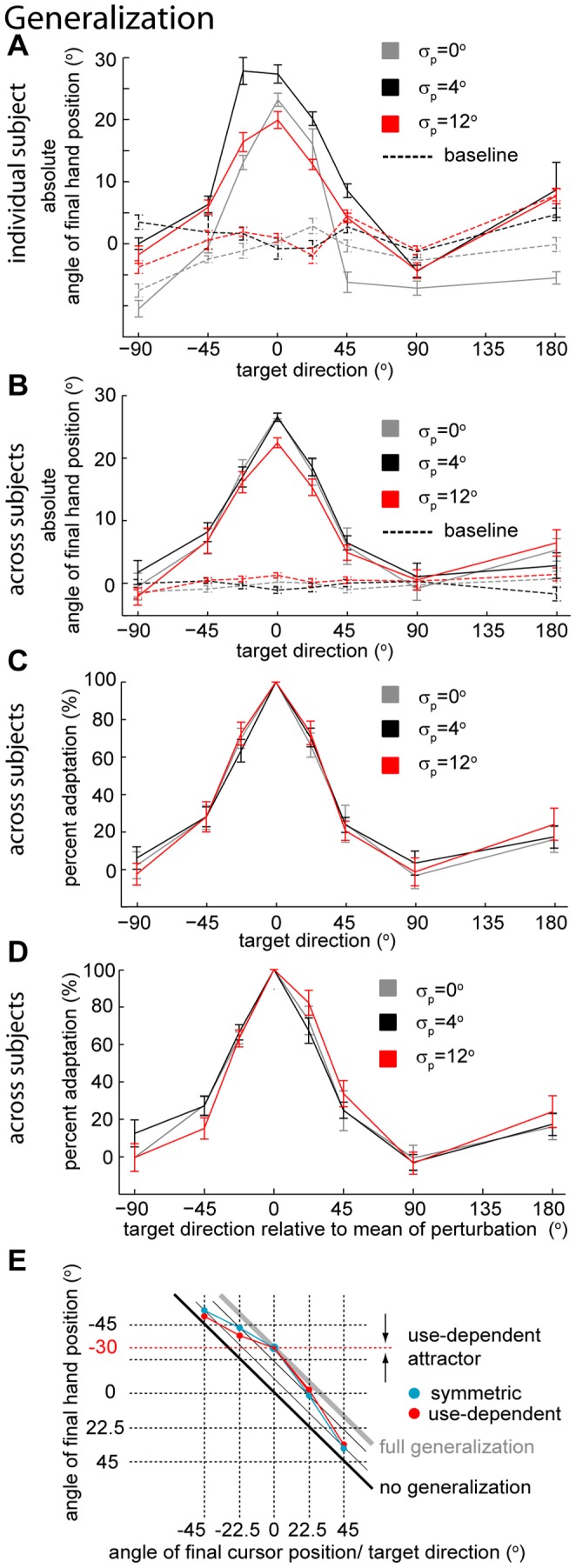
Generalization under different variance conditions. **A**) Baseline and generalization of the mean (±SEM) of a perturbation for a typical subject as measured by the absolute angle of final hand position relative to the target. Solid lines are generalization patterns after learning and dashed lines denote the pre-training (baseline) results. **B**) Average generalization (±SEM) across subjects. Solid lines denote generalization patterns after learning and dashed lines denote the pre-training (baseline) results. **C**) Percent adaptation (±SEM) in the generalizing directions relative to the learning direction. **D**) Percent adaptation (±SEM) in the generalizing directions relative to the learning direction after correcting for the sign of the mean of the perturbation; blocks with −30° mean have the target directions (x-axis) reflected relative to the learning direction. **E**) Diagram illustrating the direction of an asymmetry caused by used-dependent learning. The blue curve denotes a symmetric, local generalization pattern - without used-dependent learning. If there is used-dependent learning, hand movements in trials towards other targets would be attracted towards the direction to which the hand moved during the learning block (dashed red line). This effect would predict an asymmetry with the same side as the one observed in Fig. 3D.

Once subjects learn the perturbation in one direction we assess how this learned perturbation generalizes. Using the average final hand position during movements to the testing directions as a measure of generalization, we found that the generalization patterns are local in all three variance conditions (**Fig. 3A–C**). This is in line with Krakauer et al. [12] whose main condition was essentially identical to our 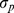 = 0° condition. Given that different subjects have different baseline biases and the amount of learning changes depending on subject and condition, we subtracted the baseline biases and normalized the generalization by the amount of learning in the learning direction – we call this measure the *percent adaptation* relative to the learning direction (**Fig. 3C**, see Methods). Despite the fact that uncertainty influenced the rate and amount of adaptation, we did not find a difference between the generalization curves in the three conditions in the absolute angle of final hand position (**Fig. 3B**) (F_2,210_ = 1.06, *p* = 0.36, two-way repeated measures ANOVA) or in the percent adaptation relative to the learning direction (**Fig. 3C**) (F_2,210_ = 0.11, *p* = 0.89, two-way repeated measures ANOVA). We also did not find a significant interaction between uncertainty levels and target angle either in the absolute angle of final hand position (F_14,210_ = 1.31, p = 0.20, two-way repeated measures ANOVA) or in the percent adaptation relative to the learning direction (F_14,210_ = 0.63, p = 0.84, two-way repeated measures ANOVA). These results suggest that the generalization pattern is independent of the uncertainty about the perturbation.

With the exception of the transformed sign of the angle of final hand position (for the measures absolute angle and percent adaptation), we have thus far ignored the sign of the perturbation (+30° or −30°) in our analysis. We can take the sign of the perturbation it into account by reflecting the target directions (x-axis in **Fig. 3A–C**) of the generalization data relative to the learning target direction for those blocks that had a −30° as mean of the distribution of perturbations. Given that all sixteen subjects are right-handed, by ignoring the sign of the mean of the distribution of perturbations while combining the data from the different subjects we expect to detect biomechanical biases that could eventually scale with the level of variability but independently of the sign of the perturbation (**Fig. 3B–C**). On the other hand if we take into account the sign of the perturbation we test for influences of angular direction of the mean of the perturbations on generalization and how these might eventually scale with uncertainty (**Fig. 3D**, see Methods for details).

When we combine the data across subjects after reflecting of the target directions according to the sign of the mean of the perturbation the generalization data, we observe an asymmetry in the generalization (**Fig. 3D**). Although we cannot reject the null hypothesis of no effect of uncertainty in the three conditions either in the absolute angle of final hand position (F_2,210_ = 1.06, *p* = 0.35, two-way repeated measures ANOVA) or in the percent adaptation relative to the learning direction (F_2,210_ = 0.11, *p* = 0.89, two-way repeated measures ANOVA), the interaction between uncertainty level and target direction appears to be significant in both in the absolute angle of final hand position (F_14,210_ = 2.06, *p* = 0.016, two-way repeated measures ANOVA) and in the percent adaptation relative to the learning direction (F_14,210_ = 1.95, *p* = 0.023, two-way repeated measures ANOVA). The direction that corresponds to maximum generalization (determined by fitting a raised von Mises-like function to each subject and condition data, see Methods for details) is not significantly different from zero for the lower uncertainty conditions (p = 0.07 and p = 0.27, for 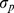 = 0° and 4°, respectively; one-sided t-test), but it is significantly different from zero for 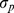 = 12° (p = 0.001, one-sided t-test). Even though it is not consistent with the amounts of uncertainty, there appears to be a weak deviation from a symmetric generalization curve.

One possible explanation for the weak asymmetry that we found is use-dependent learning [33], [34], [35]. Under this hypothesis, subjects will tend to bias their reaching towards highly repeated movements. Hand movements during the testing trials would be attracted to the direction in which the hand moved during learning. To determine whether or not use-dependent learning could account for the observed asymmetry, we first plotted a hypothetical symmetric generalization curve - the angle of final hand position (relative to the angle of the learning target) as a function of target direction (**Fig. 3E** **blue dots**) for a perturbation with mean of +30°. Use dependent learning is expected to bias these symmetric movements towards the hand movements during learning (**Fig. 3E** **red dots**). It is difficult to quantify this small effect exactly, but we observe that use-dependent learning is consistent with the direction of asymmetry that we see in our data.

To check for more subtle differences in generalization we estimated the width of the generalization curve for each individual subject and uncertainty condition (determined by fitting a raised von Mises-like function, see Methods for details). For 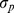 = 0°, 4° and 12° we found generalization widths of 27.0±2.2, 24.0±1.1 and 25.4±1.3 (mean±SEM, across subjects), respectively. We could not conclude that higher uncertainty corresponds to wider generalization for any of the 3 pair-wise comparisons (p = 0.79, p = 0.13 and p = 0.92 for 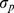 = 12 vs 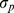 = 0°, 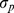 = 12° vs 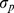 = 4° and 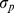 = 4° vs 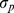 = 0°, respectively; one-sided paired t-test). These results suggest that the width of generalization of the mean of a noisy visuomotor rotation does not depend on the level of uncertainty in the perturbation.

Finally we did a post-hoc power analysis to compute the minimum detectable effect size (see Methods for details). The rationale behind this kind of analysis is that there may be a difference in generalization widths and that we did not observe it by chance or because the effect size is small over the range of noise levels used here. We computed how big the effect size should be for us to have a high expectation of observing it using a one-sided paired t-test with significance level of 0.05. We determined that we would expect a probability higher than 0.95 of observing a significant difference in the generalization widths, i.e. we would have had sufficient power to detect an effect, if the effect sizes (generalization widths) were higher than 8.5°, 5.7° and 8.2° for the 12° condition relative to 0°, the 12° relative to 4°, and 4° relative to 0°, respectively. Hence we would expect to observe a significant difference in the generalization widths even if the effect size was relatively small.

## Discussion

Here we extended traditional movement generalization studies by examining how generalization following learning of a visuomotor rotation is affected by the introduction of trial-by-trial variability. We found that generalization about the mean of a visuomotor rotation is largely unaffected when the perturbation is variable – generalization was local under three different variance conditions. Adaptation is slower and less complete with increased variance level but the width of generalization is unaffected.

We could have expected to see differences in generalization widths. Narrower or broader generalization could both have been justified based on normative arguments or under certain assumptions about the how uncertainty affects overlapping neural representation of movement. Furthermore, several previous experiments have shown that generalization widths and patterns are neither universally uniform nor immune to changes in experimental conditions. Even though the width of generalization seems to be consistent across tasks such as reaching and wrist tilting [12], [30], different kinds of perturbations show wider generalization; for example, gain perturbations in center-out reaches appear to generalize globally [12]. Also, studies that manipulate experimental conditions, such as the complexity of the perturbation [5] show changes in width of generalization. Moreover, uncertainty has been shown to affect learning [31], [36] and retention [36], in particular learning of visuomotor rotations [18], [37]. As uncertainty is important for all of these other aspects of motor learning, it may well affect generalization patterns as well. Here we have shown that generalization width for visuomotor rotations is not affected by changes in variability at least not up to 12 degrees of standard deviation.

A number of models have been proposed for how the nervous system might represent and manipulate probability distributions and uncertainty [23], [38], [39], [40], [41], [42], [43], [44], [45]. Generally in these models, the probability distribution over the set of expected perturbations or other environmental variable based on past experience is called the *prior*. After combining the prior expectations with new incoming sensory information – the *likelihood* - a new probability distribution is computed – the *posterior*. By manipulating the variance of stochastic perturbations we are modifying the variance of the prior and can alter how much subjects rely on new sensory information during single reaches [17]. However, depending on how these distributions are represented by a given neural model and precise assumptions about the neural basis of generalization, these models will make different predictions about generalization behavior.

Some models of neural representation [22], [23] explicitly propose an encoding scheme where tuning curves become wider with increasing uncertainty. Under these models neural tuning becomes broader due to the fact that neurons are receiving uncertain input [23] or because they are optimizing the representation of the prior distribution itself with narrowly tuned neurons representing more common directions/orientations [22]. Analogous models applied to movement direction would predict that higher uncertainty would lead to broader tuning curves. There is also some experimental data suggesting that individual neurons and populations of neurons are sensitive to changes in uncertainty. Receptive fields in the cat’s retina, for instance, become wider with decreasing light levels [24] and populations of neurons in pre-motor cortex appear to be able to represent uncertainty in reach plans [25]. However, there is still relatively limited experimental evidence to constrain these models of the neural representation of uncertainty, particularly in the movement related brain areas. While many electrophysiological experiments have probed how single neurons represent movement-related variables such as hand-direction, speed, or muscle activity [46], [47], [48], [49], [50] and even how neural responses change during adaptation to visuomotor rotations [3], relatively little is known about how neural activity changes in the presence of sensorimotor uncertainty (but see [25], [51], [52], [53]).

If it is true that the width of generalization curves reflects the tuning widths of the neurons, we did not find signs of such broadening in our generalization study. Importantly, there are three natural interpretations of this result. It could be that our study failed to see the effect because we did not have the necessary statistical power. However, with 16 subjects we ran far more subjects than most movement studies. Also, our power analysis revealed that we should have seen even relatively small effects of broadening; therefore it seems unlikely that this effect exists and we were unable to observe it. Another possibility is that theories that predict broadening of tuning curves are wrong, or at least do not apply to simple targeted reaching movements. However, none of the theories that deal with the representation of uncertainty explicitly mention their predictions of generalization and (third interpretation) generalization may be related to underlying neural representations in a more complex way than generally assumed in motor control research [4], [5], [6], [12].

We have found weak signs that generalization curves are slightly asymmetric. Use-dependent learning, where subjects are biased to move in a way that is similar to how they have been moving previously is one of the newly emerging insights in computational motor control [33], [34], [35]. These theories would suggest biases towards the typical direction of hand movement. We find that this is consistent with the weak asymmetry that we found in the generalization curves. Furthermore it is also a potential explanation for the commonly observed adaptation at 180° [6], [12], [30], since movements in this direction are similar to movements returning from the learning target to the center of the working space. Future research would be necessary to clarify which factors give rise to this asymmetry. For example, this asymmetry may disappear if perturbations are introduced in a gradual manner or if limb mechanics are controlled in more detail.

For some subjects the simplicity of the task and the salience of the perturbations led to cognitive strategies that may have introduced noise in the measurements. As such, we found relatively high variability across subjects. Gradually introduced perturbations have been shown to lead to a more complete adaptation and larger aftereffects [37], [54], [55]. It would be interesting to test if slowly introduced perturbations would reduce the subject-by-subject variance and even have some effect in the generalization widths.

The focus both in behavioral as well as in electrophysiological studies in motor control has been on the generalization and representation of perturbations without any trial-by-trial variability. While uncertainty has been shown to be important in many behavioral settings, variability does not appear to change generalization curves during visuomotor rotation. Variability does affect learning, however, and understanding how variability affects generalization in other tasks should provide some insight into the neural representations of uncertainty and movement.

## Materials and Methods

### Ethics Statement

The experimental protocol was approved by the Northwestern University Institutional Review Board and is in accordance with the Northwestern University Institutional Review Board’s policy statement on the use of human subjects in experiments. Written informed consent was obtained from all participants. The Institutional Review Board of Northwestern University approved the study.

### Experimental Protocol

Sixteen right-handed healthy subjects (5 male, 11 female; aged 27±3.2 years) participated in the experiment. All were naive to the purpose of the experiment, and were paid according to their performance. Subjects made center-out reaches in an approximately 150×150 mm central region of a 100 cm×70 cm workspace. They controlled the position of a cursor with their right index finger, which was recorded using an Optotrak 3D Investigator Motion Capture System. A projector and mirror system was calibrated such that visual feedback was perceived as being in the movement plane (**Fig. 1A**), and the subject’s view of their hand was blocked by the mirror.

The task was designed to measure how subjects generalize the mean of a noisy visuomotor rotation, that is, how a perturbation learned during movements in one direction affects subsequent movements in other, test directions. This experiment extends a previous paradigm that allows measurement of generalization about a fixed perturbation [12] to include stochastic perturbations.

Subjects were instructed to make center-out reaches into a certain direction (the learning direction) until they adapted to the perturbations/rotations. During this period subjects were given endpoint feedback - that is, the final position of the hidden cursor was displayed - and were eventually able to correct endpoint errors in the learning direction. Afterwards, they were instructed to make movements into other directions (the generalizing directions) in order to measure the generalization pattern of the learned mean of the perturbation. Generalization of the mean was assessed by analyzing their average reaching direction for each target.

The learning direction was randomly sampled from one of the 4 diagonal directions and generalization was measured in 7 directions: 180°, ±90°, ±45° and ±25° from the learning direction (**Fig. 1C**). Subjects controlled the position of a red circle, the cursor (∼3 mm radius), with their right index finger. Except for the first familiarization trials the position of the cursor was hidden. Subjects were instructed to make radial reaches from a central blue circle, the starting circle (∼6 mm radius) to one of 8 yellow circles, the targets (∼6 mm radius). Targets were all displayed at a distance of 72 mm from the central blue circle. 300 ms after positioning the cursor over the blue circle, the cursor disappeared, one of the eight targets appeared and subjects had to reach it. On some of the trials the final position of the cursor was displayed for 500 ms (endpoint feedback). The final position of the cursor was defined as the first position of the cursor when its center was at a distance greater than 72 mm from the center of the starting circle. If the reach was successful, that is, if the center of the red cursor was inside the target then the target turned white and subjects were rewarded by having a point added to their score. If a successful reach happened in those trials where no information was provided about the success of the reach (no endpoint feedback) then a point was added to a hidden score. To initiate the next trial, subjects had to reposition the cursor in the starting blue circle. Except for the familiarization trials where the cursor was always visible, the cursor was visible only within a distance of 10 mm from the center of the starting blue circle. Since some subjects have difficulty finding their way back to the starting blue circle, 4 seconds after the previous trial was over, the cursor flashed every second for 50 ms to allow subjects to find the starting position.

We measured generalization of the learned mean for a rotation of ±30° under three variability levels. Each trial, noise was added to the visuomotor rotation drawn from a Gaussian with a standard deviation of 0°, 4° or 12°. The standard deviation of 0° reproduces previous experiments that measured the generalization pattern of a deterministic visuomotor rotation [12].

The experiment was divided into three blocks of 560 trials (**Fig. 1B** **)**. Blocks differed in the level of variance and were pseudo-randomized. Each block was composed of 4 sub-blocks: *Familiarization*, *Baseline*, *Learning* and *Testing*. No rotation was imposed during the familiarization and baseline blocks. In all cases, the maximum time to complete each trial was 4 seconds and the minimum time 40 ms. If any of these times was violated the trial was restarted.

#### Familiarization

During the first half (40 trials, 5 movements to each target) of the familiarization sub-block the cursor was always visible. During the second half (40 trials, 5 movements to each target) only endpoint position was displayed.

#### Baseline

This sub-block was used to measure the baseline (80 trials, 10 movements to each target). These reaches were made under the same conditions as the second half of the familiarization block – endpoint feedback was provided in all trials and no perturbation was applied to the cursor.

#### Learning

Subjects completed 240 trials of movements towards a single learning target with only endpoint feedback. The cursor was rotated relative to hand position.

#### Testing

The testing sub-block was composed of 160 trials. In order to prevent de-adaptation to the perturbation, the learning target was revisited at least twice every 4 trials; every sequence of 4 trials consisted of two reaches towards the learning target and two reaches towards any two of the 8 targets. Targets were chosen pseudo-randomly so that there were 10 reaches total towards each of the generalizing directions. Endpoint feedback is provided only in the learning direction trials. During these trials towards the learning direction the perturbations applied to the cursor position were sampled from the same distribution used in the learning block.

### Data Analysis

Final hand position angle gives us a measure of the subject’s estimation of the perturbation. For each trial we computed the final hand position by averaging the last data point before the hand goes beyond a distance of 72 mm – the target radial distance – from the center of the starting circle and the first data point after that. Notice that final hand position is well defined for every trial since trials were restarted whenever the subject did not go beyond a distance of 72 mm.

#### Absolute final hand position and percent adaptation

Since the sign of the mean of the distribution of perturbation was randomly chosen for each block and each subject, we normalized the angle of final hand position according to the sign of mean of the perturbations so that the average final hand position angle in the learning direction was positive for every block; this was done by multiplying by −1 the angle of final hand position when the mean of the distribution of perturbations was positive (+30 degrees). We call this measure the *absolute final hand position.* We measured the baseline movement biases, 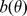, and the learned and generalized means, 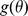, by considering the average absolute angle of final hand position (**Fig. 2**). Specifically, 
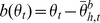
 and 
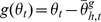
, where 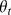 is target direction, 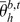 and 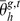 are average absolute angles of final hand position in trials towards target 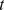 during baseline and testing, respectively. Using this information we can compute the *percent adaptation*, that is, the difference between testing and baseline in the each direction relative to the learning direction 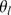 (**Fig. 3C**):
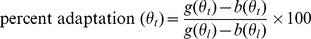



Notice that a positive absolute angle of final hand position or percent adaptation corresponds to a hand movement that counteracts the mean of the distribution of perturbations. We use one of these two measures in every figure and analysis (with the exception of **Fig. 2A** and **3E** where the true sign of final hand angle is displayed).

#### Time-scales of learning

To compute the time scales and amount of adaptation we considered only the first block of learning for each subject (n = 8, n = 4 and n = 4 for 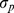 = 0°, 4° and 12°, respectively). We then fitted exponential learning curves that were constrained to start at zero. We used bootstrapping over trials to determine the p-value for the differences between the timescales of learning and between adaptation at end of the learning sub-blocks.

#### Correcting for the sign of the mean of the perturbation

For part of the analysis (**Fig. 3D**) we wanted to take into account the fact that, for some of the blocks, the mean of the imposed perturbation had negative sign (−30°). This was done with the objective of searching for aspects of generalization that could depend on the sign of the imposed perturbation. We did the correction by reflecting the target directions relative to the learning target direction; if we set the learning target direction, 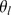 to be zero, then the corrected generalization function 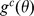 is defined as: 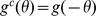.

#### Width of generalization

To determine the generalization width we used raised von Mises-like (circular Gaussian) functions:
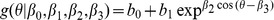
(1)where 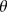 is target direction. We fitted these functions to each individual percent adaptation generalization. We used 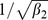 as the estimate of generalization width. We excluded two subjects from this analysis because the estimated width of their generalization in at least one of the uncertainty conditions was more than 10 standard deviations away from the mean of the remaining subjects’ widths for that uncertainty condition.

#### Peak of generalization

To determine if there is a consistent asymmetry in the generalization pattern, we determined, for each subject and each uncertainty condition, the angle of maximum generalization given by the parameter 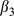 in Equation 1. The sign of the parameter was corrected for the sign of the mean of the perturbation, more specifically, we multiplied 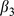 by the sign of the mean of the perturbation.

#### Effect size

To compute the minimum effect size, 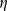, that would have been required for detecting an significant effect with probability above 0.95 using a two-sample one-sided paired t-test at a significance level of 0.05, we used the standard minimum detectable effect formula (for e.g. see [56]).
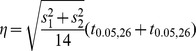
where 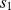 and 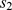 are the estimated variances of widths for each uncertainty condition and 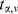 represents the value of the inverse of the cumulative t-student distribution with 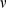 degrees of freedom at 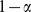.

## References

[1] Christopoulos VN, Schrater PR (2009) Grasping objects with environmentally induced position uncertainty. PLoS computational biology 5: e1000538. 19834543 10.1371/journal.pcbi.1000538 PMC2756623

[2] Shadmehr R (2004) Generalization as a behavioral window to the neural mechanisms of learning internal models. Human movement science 23: 543–568. 15589621 10.1016/j.humov.2004.04.003 PMC2722915

[3] Paz R, Boraud T, Natan C, Bergman H, Vaadia E (2003) Preparatory activity in motor cortex reflects learning of local visuomotor skills. Nature neuroscience 6: 882–890. 12872127 10.1038/nn1097

[4] Thoroughman K, Shadmehr R (2000) Learning of action through adaptive combination of motor primitives. Nature 407: 742–747. 11048720 10.1038/35037588 PMC2556237

[5] Thoroughman K, Taylor J (2005) Rapid reshaping of human motor generalization. Journal of Neuroscience 25: 8948–8953. 16192385 10.1523/JNEUROSCI.1771-05.2005 PMC6725605

[6] Donchin O, Francis JT, Shadmehr R (2003) Quantifying generalization from trial-by-trial behavior of adaptive systems that learn with basis functions: theory and experiments in human motor control. The Journal of neuroscience 23: 9032–9045. 14534237 10.1523/JNEUROSCI.23-27-09032.2003 PMC6740843

[7] Hwang EJ, Smith MA, Shadmehr R (2006) Adaptation and generalization in acceleration-dependent force fields. Experimental brain research 169: 496–506. 16292640 10.1007/s00221-005-0163-2 PMC1456064

[8] Goodbody S, Wolpert D (1998) Temporal and amplitude generalization in motor learning. Journal of Neurophysiology 79: 1825–1838. 9535951 10.1152/jn.1998.79.4.1825

[9] Shadmehr R, Moussavi Z (2000) Spatial generalization from learning dynamics of reaching movements. Journal of Neuroscience 20: 7807–7815. 11027245 10.1523/JNEUROSCI.20-20-07807.2000 PMC6772893

[10] Pearson TS, Krakauer JW, Mazzoni P (2010) Learning not to generalize: modular adaptation of visuomotor gain. Journal of neurophysiology 103: 2938–2952. 20357068 10.1152/jn.01089.2009 PMC2888232

[11] Shadmehr R, Mussa-Ivaldi F (1994) Adaptive representation of dynamics during learning of a motor task. Journal of Neuroscience 14: 3208–3224. 8182467 10.1523/JNEUROSCI.14-05-03208.1994 PMC6577492

[12] Krakauer J, Pine Z, Ghilardi M, Ghez C (2000) Learning of visuomotor transformations for vectorial planning of reaching trajectories. Journal of Neuroscience 20: 8916–8924. 11102502 10.1523/JNEUROSCI.20-23-08916.2000 PMC6773094

[13] Ghahramani Z, Wolpert DM, Jordan MI (1996) Generalization to local remappings of the visuomotor coordinate transformation. The Journal of neuroscience 16: 7085–7096. 8824344 10.1523/JNEUROSCI.16-21-07085.1996 PMC6579263

[14] Mattar A, Ostry D (2007) Modifiability of generalization in dynamics learning. Journal of neurophysiology 98: 3321–3329. 17928561 10.1152/jn.00576.2007

[15] Taylor JA, Ivry RB (2011) Feedback-dependent generalization of visuomotor adaptation. Advances in Computational Motor Control 10.

[16] Tassinari H, Hudson T, Landy M (2006) Combining priors and noisy visual cues in a rapid pointing task. Journal of Neuroscience 26: 10154–10163. 17021171 10.1523/JNEUROSCI.2779-06.2006 PMC6674625

[17] Körding K, Wolpert D (2004) Bayesian integration in sensorimotor learning. Nature 427: 244–247. 14724638 10.1038/nature02169

[18] Saijo N, Gomi H (2012) Effect of visuomotor-map uncertainty on visuomotor adaptation. Journal of neurophysiology 107: 1576–1585. 22190631 10.1152/jn.00204.2011

[19] Wei K, Körding K (2010) Uncertainty of feedback and state estimation determines the speed of motor adaptation. Frontiers in computational neuroscience 4: 11. 20485466 10.3389/fncom.2010.00011 PMC2871692

[20] van Beers RJ (2009) Motor learning is optimally tuned to the properties of motor noise. Neuron 63: 406–417. 19679079 10.1016/j.neuron.2009.06.025

[21] Harris CM, Wolpert DM (1998) Signal-dependent noise determines motor planning. Nature 394: 780–784. 9723616 10.1038/29528

[22] Girshick AR, Landy MS, Simoncelli EP (2011) Cardinal rules: visual orientation perception reflects knowledge of environmental statistics. Nature neuroscience 14: 926–932. 21642976 10.1038/nn.2831 PMC3125404

[23] Zemel R, Dayan P, Pouget A (1998) Probabilistic interpretation of population codes. Neural computation 10: 403–430. 9472488 10.1162/089976698300017818

[24] Barlow H, Fitzhugh R, Kuffler S (1957) Change of organization in the receptive fields of the cat’s retina during dark adaptation. The Journal of physiology 137: 338–354. 13463771 10.1113/jphysiol.1957.sp005817 PMC1363009

[25] Cisek P, Kalaska J (2005) Neural correlates of reaching decisions in dorsal premotor cortex: specification of multiple direction choices and final selection of action. Neuron 45: 801–814. 15748854 10.1016/j.neuron.2005.01.027

[26] Poggio T, Bizzi E (2004) Generalization in vision and motor control. Nature 431: 768–774. 15483597 10.1038/nature03014

[27] Pouget A, Snyder LH (2000) Computational approaches to sensorimotor transformations. Nature neuroscience 3: 1192–1198. 11127837 10.1038/81469

[28] Poggio T (1990) A theory of how the brain might work. Cold Spring Harbor Symp Quant Biol 55: 899–910. 2132866 10.1101/sqb.1990.055.01.084

[29] Paz R, Nathan C, Boraud T, Bergman H, Vaadia E (2005) Acquisition and generalization of visuomotor transformations by nonhuman primates. Experimental Brain Research 161: 209–219. 15480596 10.1007/s00221-004-2061-4

[30] Fernandes HL, Albert MV, Kording KP (2011) Measuring generalization of visuomotor perturbations in wrist movements using mobile phones. PloS one 6: e20290. 21629659 10.1371/journal.pone.0020290 PMC3101241

[31] Berniker M, Voss M, Kording K, Brezina V (2010) Learning Priors for Bayesian Computations in the Nervous System. PloS one 5: e12686. 20844766 10.1371/journal.pone.0012686 PMC2937037

[32] Burge J, Ernst MO, Banks MS (2008) The statistical determinants of adaptation rate in human reaching. Journal of Vision 8: 20.21–19. 10.1167/8.4.20 PMC2684526 18484859

[33] Huang VS, Haith A, Mazzoni P, Krakauer JW (2011) Rethinking motor learning and savings in adaptation paradigms: model-free memory for successful actions combines with internal models. Neuron 70: 787–801. 21609832 10.1016/j.neuron.2011.04.012 PMC3134523

[34] Verstynen T, Sabes PN (2011) How each movement changes the next: an experimental and theoretical study of fast adaptive priors in reaching. The Journal of neuroscience 31: 10050–10059. 21734297 10.1523/JNEUROSCI.6525-10.2011 PMC3148097

[35] Diedrichsen J, White O, Newman D, Lally N (2010) Use-dependent and error-based learning of motor behaviors. The Journal of neuroscience 30: 5159–5166. 20392938 10.1523/JNEUROSCI.5406-09.2010 PMC6632748

[36] Shea CH, Kohl RM (1990) Specificity and variability of practice. Research quarterly for exercise and sport 61: 169–177. 2094928 10.1080/02701367.1990.10608671

[37] Turnham EJA, Braun DA, Wolpert DM (2012) Facilitation of learning induced by both random and gradual visuomotor task variation. Journal of Neurophysiology 107: 1111–1122. 22131385 10.1152/jn.00635.2011 PMC3289458

[38] Ma WJ (2010) Signal detection theory, uncertainty, and Poisson-like population codes. Vision Research 50: 2308–2319. 20828581 10.1016/j.visres.2010.08.035

[39] Fiser J, Berkes P, Orbán G, Lengyel M (2010) Statistically optimal perception and learning: from behavior to neural representations. Trends in cognitive sciences 14: 119–130. 20153683 10.1016/j.tics.2010.01.003 PMC2939867

[40] Sahani M, Dayan P (2003) Doubly distributional population codes: simultaneous representation of uncertainty and multiplicity. Neural Computation 15: 2255–2279. 14511521 10.1162/089976603322362356

[41] Deneve S (2008) Bayesian spiking neurons I: inference. Neural computation 20: 91–117. 18045002 10.1162/neco.2008.20.1.91

[42] Ma W, Beck J, Latham P, Pouget A (2006) Bayesian inference with probabilistic population codes. Nature Neuroscience 9: 1432–1438. 17057707 10.1038/nn1790

[43] Hoyer PO, Hyvarinen A (2003) Interpreting neural response variability as Monte Carlo sampling of the posterior. Advances in neural information processing systems: 293–300.

[44] Hinton GE, Sejnowski TJ. Optimal perceptual inference; 1983. IEEE Press Piscataway, NJ. 448–453.

[45] Berkes P, Orbán G, Lengyel M, Fiser J (2011) Spontaneous cortical activity reveals hallmarks of an optimal internal model of the environment. Science 331: 83. 21212356 10.1126/science.1195870 PMC3065813

[46] Georgopoulos A, Ashe J, Smyrnis N, Taira M (1992) The motor cortex and the coding of force. Science 256: 1692–1695. 1609282 10.1126/science.256.5064.1692

[47] Sergio L, Hamel-Paquet C, Kalaska J (2005) Motor cortex neural correlates of output kinematics and kinetics during isometric-force and arm-reaching tasks. Journal of Neurophysiology 94: 2353. 15888522 10.1152/jn.00989.2004

[48] Graham K, Moore K, Cabel D, Gribble P, Cisek P, et al (2003) Kinematics and kinetics of multijoint reaching in nonhuman primates. Journal of Neurophysiology 89: 2667–2677. 12612006 10.1152/jn.00742.2002

[49] Kakei S, Hoffman D, Strick P (1999) Muscle and movement representations in the primary motor cortex. Science 285: 2136–2139. 10497133 10.1126/science.285.5436.2136

[50] Moran D, Schwartz A (1999) Motor cortical representation of speed and direction during reaching. Journal of Neurophysiology 82: 2676–2692. 10561437 10.1152/jn.1999.82.5.2676

[51] Cisek P, Kalaska J (2002) Simultaneous encoding of multiple potential reach directions in dorsal premotor cortex. Journal of Neurophysiology 87: 1149–1154. 11826082 10.1152/jn.00443.2001

[52] Rickert J, Riehle A, Aertsen A, Rotter S, Nawrot M (2009) Dynamic encoding of movement direction in motor cortical neurons. Journal of Neuroscience 29: 13870–13882. 19889998 10.1523/JNEUROSCI.5441-08.2009 PMC6666727

[53] Britten K, Shadlen M, Newsome W, Movshon J (1992) The analysis of visual motion: a comparison of neuronal and psychophysical performance. Journal of Neuroscience 12: 4745–4765. 1464765 10.1523/JNEUROSCI.12-12-04745.1992 PMC6575768

[54] Kagerer FA, Contreras-Vidal J, Stelmach GE (1997) Adaptation to gradual as compared with sudden visuo-motor distortions. Experimental brain research 115: 557–561. 9262212 10.1007/pl00005727

[55] Taylor JA, Ivry RB (2011) Flexible cognitive strategies during motor learning. PLoS computational biology 7: e1001096. 21390266 10.1371/journal.pcbi.1001096 PMC3048379

[56] Zar JH (1999) Biostatistical analysis. Prentice Hall New Jersey 4.

